# Knowledge and perceptions of tuberculosis among patients in a pastoralist community in Kenya: a qualitative study

**DOI:** 10.11604/pamj.2018.30.287.14836

**Published:** 2018-08-23

**Authors:** Grace Wambura Mbuthia, Charles Owour Olungah, Tom Gesora Ondicho

**Affiliations:** 1 College of Health Sciences, Moi University, Eldoret, Kenya; 2 Institute of Anthropology Gender and African Studies, University of Nairobi, Kenya

**Keywords:** Tuberculosis, knowledge, qualitative study, Kenya

## Abstract

**Introduction:**

Tuberculosis awareness is crucial to the success of control and prevention of tuberculosis. However, the knowledge and perceptions of tuberculosis patients in rural Kenya is not well documented. The study sought to explore the knowledge and perceptions of TB patients in West Pokot County Kenya.

**Methods:**

This was a qualitative descriptive study conducted between January-March 2016. A total of 61 pulmonary tuberculosis patients took part in the study which comprised 6 focus group discussion and 15 in-depth interviews. Thematic analysis was used to analyse the data.

**Results:**

Participants perceived TB as a serious contagious disease that is hard to diagnose and treat. They attributed tuberculosis to smoking, drinking alcohol, dust, cold air, witchcraft, trauma to the chest, contact with livestock and genetic factors. They believed that TB was transmitted through casual contact with TB patients and sharing of utensils.

**Conclusion:**

The study showed a lot of misperceptions among tuberculosis patients. The tuberculosis program should heighten patient education to improve patient knowledge and put more effort to dispel misinformation about the cause and mode of transmission of the disease.

## Introduction

Tuberculosis(TB) is a major global health concern [1]. It is the most common infectious cause of mortality worldwide surpassing malaria and Human Immunodeficiency Virus/Acquired Immune Deficiency Syndrome (HIV/AIDs) [2]. According to World Health organization(WHO), lack of knowledge about TB causes underutilization of the services, delay in seeking diagnosis, and poor treatment adherence [3]. Consequently creating general awareness about TB among communities and initiating community participation in the control of the disease makeup one component of the 6 basic components of the "Stop TB Strategy" of the WHO [4]. Improving community's knowledge on TB is essential in the TB Control strategy as it shapes their health-seeking behaviour [5-7]. Several studies have shown that dearth of knowledge about the etiology, cardinal symptoms, route of transmission as well as appropriate treatment of TB may lead to delayed or inappropriate health-seeking practices, thus sustaining the transmission of the disease within the community [7-15]. According to Mondal et al (2014), although people often have a general idea of what TB is, gaps in knowledge on transmission, treatment and prevention leads to diagnostic and treatment delays among people living with TB. The author argues that patients with low knowledge about TB are less likely to seek healthcare and get diagnosed rather they often turn to self-medication and traditional healers which lead to delays in diagnosis and appropriate treatment [16]. In Ethiopia, Abebe and colleagues found that lack of awareness on TB contributed to the late presentation of suspected TB patient in the health facility [17]. While several studies done in African settings indicate that community members often have incorrect knowledge about the cause and transmission of TB [15, 17-20], the knowledge and perceptions among TB patients has not been well explored. The current study therefore, focused on TB patients on treatment. The study sought to assess the knowledge and perception of TB among TB patients in 4 health facilities in West Pokot County, Kenya.

## Methods

**Study setting**: This was a facility-based study carried out in four hospitals offering TB services in West Pokot County, Kenya. The County is located in the Rift Valley and lies within Longitudes 34º 47´ and 35º 49´ East and Latitude 10º 10´ and 30º 40´ North. The County has a population of 512,690 people (2009 census) and an area of 9,169.4 km^2^. It is less developed with poor infrastructure and the inhabitants are mainly Pokots. Approximately 80% of the County is arid or semiarid and 60% of the inhabitants are nomadic pastoralists while the rest of the population are agro-pastoralists [21]. There are 21 TB treatment sites in the county [22]. The main government treatment sites are one County hospital and three Sub County hospitals included in this study. The rest include faith-based health facilities, health centres and dispensaries. West Pokot County had a higher TB case notification rate of 225 per 100,000 population compared to the national rate of 217 per 100,000 population [22].

**Study design**: This was a qualitative descriptive study conducted between January-March 2016 using focus group discussion (FGDs) and in-depth interviews (IDIs).

**Participant recruitment and sampling**: The study participants composed of 61 pulmonary tuberculosis patients receiving treatment from four health facilities in West Pokot County. Only confirmed adult TB cases on treatment were included in the study. The mentally ill and patients who had not completed two weeks of treatment were excluded from the study due to the infectious nature of the disease. With the guidance of the nurses at the TB clinics, participants who met the inclusion criteria were purposively selected and briefed about the study and were booked for the interviews during their appointment dates.

### Data collection methods

**In-depth interviews**: In-depth interviews were the primary method used to collect data on patient knowledge and perceptions. The interviews were done at the TB clinics and lasted for 45-60 minutes. A semi-structured interview guide was used to collect the information. The interviews were conducted in Kiswahili language at the TB clinic and were tape recorded and later transcribed verbatim [23]. The concept of data saturation [24] was used to guide the number of IDIs conducted. Data collection was stopped at 15 IDIs. The Kiswahili tape-recorded interviews were translated into English and transcribed in English.

**Focus group discussion**: We conducted six FGDs (3 with males and 3 with females) each comprised of 6-10 participants. Compared to individual interviews, group interaction allows participants to agree and disagree thereby stimulating richer responses which aid in revealing the respondent's real perceptions on the subject of interest [25]. Gathering ideas and cultural beliefs surrounding TB was possible through this method of data collection. The FGDs were constituted based on gender. A semi structured FGD guide was used to collect the data. The FGDs were conducted in Kiswahili language and each lasted for 60 to 90 minutes. Each FGD was audio-recorded and later transcribed verbatim [23]. The concept of theoretical saturation was used to ensure no new conceptual information was emerging from further discussions [24]. Data saturation was reached at 6 FGDs.

**Data processing and analysis**: The FGDs and IDIs were transcribed by the researcher. Transcripts were analysed with the aid of the N-vivo (version 11). Data collection and data analysis were done concurrently. Thematic analysis was done by reading through the transcript multiple times and identifying, coding and categorizing meaningful patterns into themes and sub-themes.

**Ethical considerations**: The research proposal was approved by Moi University College of Health Sciences/Moi Teaching and Referral Hospital Institutional Research and Ethics Committee (Formal Approval Number: IREC 0001349). Participants were all briefed on the study and each respondent asked to sign an informed consent form without coercion. Participants allowed the audio recording during data collection and were assured of confidentiality and anonymity for any information given.

## Results

**Socio-demographic characteristics of the participants**: A total of 61 participants were enrolled in the study. The median age of the participants was 38 (range 27-61 years). A total of 29(47.5 %) were females while 32(52.5%) were males. Out of the 61 participants, 46(75%) participated in the FGD while 15(25%) participated in the in-depth interviews. About 27(44%) of the participants had no formal education while 21(35%) and 13(21%) had attained primary and secondary education respectively. Majority 34(56%) were pastoralist while 11(18%) and 10(17%) indicated business and formal employment as their main source of income. The rest (9%) indicated they had no source of income.

**Knowledge and perceptions of TB**: Tuberculosis was commonly referred to as "TB" by the study participants. When asked the local name for the disease, the participants reported that among the Pokot community, TB was known as Semewo takat meaning disease of the chest. The findings revealed that TB patients had different perceptions about TB. There were five themes namely: curable disease, serious illness hard to diagnose and treat, contagious disease, a disease caused by a germ, and misconceptions. The misconception theme had 2 sub-themes.

**Curable disease**: The data revealed that patients correctly perceived TB as a curable disease. They were also cognisant to the fact that for one to be cured of TB they needed to adhere to treatment for a long period of time. The fact that the patients were aware of efficacious drugs against TB made it less stressful for the patients to learn that they were suffering from TB. Two participants had this to say; *"…TB is curable but one has to be persistent and take drugs for a very long time…" (Female 30 years). "I wasn't scared at all because TB is a disease that is treatable as well as curable." (Male 32 years).* Patient's previous experiences were key in shaping their perceptions about TB. One patient expressed how devastated he was to learn that he had TB which according to him was a fatal disease. He believed that TB can be fatal particularly if one does not seek and adhere to the doctor's instruction. His experience of having witnessed a patient die of TB in his village made him worried about his illness. However, he found relief after the nurse explained that TB was curable and showed him evidence of people who had been treated and cured of TB. This implies the importance of patient education by the health workers in creating awareness on facts about tuberculosis. This was illustrated by a male patient in his response to the question, how did you feel after being told you were suffering from TB? *"When they told me I had TB I got scared that I might die and leave my children without a caregiver…but the nurse reassured me that it is a treatable disease and that I will get cured when I take the drugs well. I was scared because I used to have a neighbour who had TB, he was told to stop taking alcohol and smoking but he continued until he died. I was encouraged by the doctor who gave me an example of 5 people who had TB and yet they recovered…this gave me hope" (Male 28 years).*

The participants alluded to the fact that for one to be cured of TB they needed to adhere to treatment for a long period of time. Majority of the participants viewed this as a major challenge in dealing with the disease. However, some of the patients indicated having abandoned treatment prematurely after their health had improved. This only resulted to more suffering as the disease recurred and patient forced to restart a full treatment regimen. This may be attributed to lack of knowledge about TB treatment among some of the TB patients in West Pokot County. This was alluded to by the participants in their narratives. *"The problem with TB is that you have to take treatment for a very long time and sometimes you give up. Personally I swallowed the drugs until I felt I had recovered. I swallowed for one and half month and I felt I had Improve and so I stopped the medications" (Female FGD two). "Even me I swallowed the drugs for some time until I had Improved and so I stopped the treatment. But after one and a half years I had severe cough and came back here…" (Female FGD two).*

**Contagious disease**: Majority of the participants perceived TB as a contagious disease that can easily spread from one person to the other. Some of them correctly indicated that TB is an airborne disease and emphasised the need to observe cough etiquette to prevent transmission. However most of the participants held a lot of misperceptions on TB transmission. This is illustrated below in the misconceptions theme. Some of the participants had this to say; *"I have heard that TB is transmitted to another person through coughing. The doctors here tell us to cover our mouth when we are coughing so that we don't pass the disease to the other people" (Female FGD one). "TB can be transmitted through air when you cough or sharing utensils." (Male 45 years).*

**A serious disease hard to diagnose and treat**: Most patients thought that TB was a severe disease that mainly affect the chest and has an insidious onset which makes it hard to diagnose. Majority were concerned about the frustration one has to go through before getting the correct diagnosis and treatment. Due to the onset of symptoms which mimic other respiratory tract infections, patients were often treated with different medications without improvement. This was illustrated by the following sentiments; *"…with TB life is complicated, some of us have gone to so many hospitals before being told the problem is TB" (Female FGD three). "TB is a bad disease it hides in the body and it is not easy to know that you are suffering from TB. Because it starts just like a common cold with a cough…" (Female 49 years).* The participants perceived TB as a source of great suffering to the patient. Several participants agonised how difficult it was to go through the experience of having TB. They recounted how TB caused them a lot of pain and discomfort which left them very weak and unable to lead a comfortable life. Some of the participants had this to say; *"When you have TB you suffer a lot and you experience a lot of chest pains and you also cough a lot. It is a bad disease that sucks the body making you lose weight… It makes someone to vomit a lot and loss appetite and this makes you very weak" (Male 28 years). "TB is a serious disease that makes you lose weight, "inakunyonya kama kupe" (it sucks you like a tick) it sucks you until you become very weak and everyone can notice you are unhealthy" (Female FGD one).* The participants were cognizant to the fact that TB is fatal without treatment. They termed TB as "a very bad disease" which require medical attention. According to the participants, if one has TB they should seek the right treatment from what they referred to as "big hospitals" meaning the County or Sub-county hospitals. *“When one has that disease he should go to the hospital because it is a very bad disease" (Male FGD one). "TB is a bad disease that can finish you and the best thing is to look for treatment in a big hospital like this one so that your problem is discovered early and cured" (Male 28 years).*

**TB is caused by germ**: One of the probing questions on knowledge about TB in both narrative guide and focus group discussion guide was "What causes TB"? The data from both the narrative and the focus group discussions showed that participants had different explanations as to what causes TB. Only 2 participants in the focus group discussion indicated that TB is caused by germ or bacteria. The rest of the participants held a lot of misconceptions about the cause of TB as discussed below in the misconceptions theme.

**Misconceptions about TB**: The participants had a lot of false beliefs and myths concerning the cause and transmission of tuberculosis.

**Notions on the cause of TB**: The participants indicated that TB was a hereditary disease. According to them, this was the explanation for having more than one person from the same family suffer from TB. Although most of them termed TB as a contagious disease they did not attribute transmission to be the reason why members of the same family could suffer from tuberculosis. To some of the patients, having a member of the family suffer from TB was expected since this was an inherited disease. This was demonstrated by some of the participants who had this to say; *"TB is hereditary. It is a family disease like in my case most of the members have been treated for TB. It runs in our family. Even when they told me I had the disease I was not surprised" (Female FGD three)… if someone from your family has suffered from TB, then automatically someone else in the family will have to suffer from TB. That is, they say that there some families/clan who have had this disease from olden days and it will continue like that even in future generations" (Female FGD one).* Similarly, the participants perceived smoking and drinking alcohol as the cause of TB. The participants particular those who had the habit of drinking alcohol and smoking cigarettes found this as the only explanation as to why they acquired TB. *"TB is caused by drinking alcohol and smoking cigarettes. If you look keenly you will find those who take a lot of illicit brews get TB. I used to take alcohol and that's how I got the disease but now I have stopped" (Female FGD two).* The mistaken beliefs on the cause of TB affect the control and preventive measures the community may advocate for. According to the participants since drinking alcohol and smoking was a major cause of TB, one of the measures to reduce the burden of TB in the county was that the government should ban the consumption of illicit brew and cigarette smoking. This was illustrated by the following participant; *"TB is caused by drinking and cigarettes smoking. To reduce this problem the government should ban smoking and stop consumption of all illicit brews" (Male FGD two).* The participants attributed dusty environment and the dry weather predominant in West Pokot as the cause of the increased cases of TB in the area. According to them, TB mainly affect people who are exposed to dust due to the nature of their jobs. Some of the participants had this to say; *"It affects those people who smoke and those who work in dusty places. This place is dry and thus why we have a lot of TB" (Male FGD two). "TB is a lot in this region because of the dry weather and a lot of dust" (Female FGD one).*

Other participants felt TB was as result of both cold air and dust as indicated by one of the male respondent; *"TB is caused by dust and cold air" (Male FGD one).* For some of participants TB is a zoonotic disease that spread from the goats to humans. The participants were mainly pastoralists and believed that their interaction with the domestic animals was a source of TB. Due to animal theft, communities in the region often share room with their goats and sheep. To some of the participants this was one of the cause of tuberculosis. *…also the practice of rearing goats where some people sleep in the same room with the goats may be causing the many cases of TB" (Female FGD three). "TB is disease that affects the chest mainly and it is brought by the close contact with goats. Living in the same room with goats can bring the infection to the humans" (Male 61 years).*For others TB was as result of trauma to the chest. Some of the participants associated their illness to be as result of trauma that they had suffered at some point before the TB symptoms set in. Several patients recounted that their chest problems started after some injury to the chest which was later diagnosed as tuberculosis. A participant had this to say; *"One can get TB when you suffer from Trauma. My problem with TB started when I fell from a tree and hurt my ribs. After sometime I started coughing and thus how my problem all started" (Male FGD three).* Worse still, for other participants TB was as a result of bad omen, curse or witchcraft. One of the key questions in the FGD guide was "What are some of the traditional explanations to the causes of TB"" In response to this question a minority of the participants indicated that TB was not viewed as an infectious disease. To them TB was a curse or a bad omen that can befall anyone. *"…people say TB comes as a result of curse or a bad omen which can affect anyone…some think that it is witchcraft"" (Female FGD three).*

**Patients' notions on TB transmission**: When asked whether TB is transmissible, the participants correctly perceived TB as contagious, however majority had false notions on how TB spread from one person to the other. This is of concern since it is likely to affect the preventive and control measures adopted by the community. Participants believed that TB was transmitted through sharing of utensils. They noted that they all had their utensils set aside from the rest of the family to avoid transmitting the illness. *"The disease can be transmitted through sharing utensils. That is why it is always good to have your own cup spoon plate even cooking pots …I don't know of any other route. (Male 40 years) "If you have TB you are supposed to have your cup, plate, spoon cooking pot and even beddings isolated from the rest in the family." You must not share with the others (Female FGD two).* For others TB was transmitted through casual contact with an infected person. In order to prevent transmission they advised that an infected person should avoid associating with the rest of the family including having their own utensils and house. This often led to acts of isolation as described by some of the participants. *"When one has TB she should not shake hands with the healthy people until one completes the treatment" (Female FGD two). "TB is also transmitted when you eat and drink with a person who has TB. People with TB should have their own utensils and should not share house with the rest of the family" (Female FGD three).*

## Discussion

The study showed that, though the participants correctly perceived TB as a contagious disease that is curable they did not know the cause and the mode of transmission of tuberculosis. Participants attributed the cause of TB to genetic factors, drinking alcohol and smoking, cold air, trauma, dusty environment as well as bad omens while sharing of utensils and casual contact were seen as the main routes of transmitting TB. This is despite the fact that these were patients who were already on treatment and ought to have received TB education at the health facility. The lack of TB knowledge is of great concern as it leads to wrong opinions on control and prevention of TB thereby making it difficult to reduce the burden of TB. The current study revealed there were false beliefs and opinion about the cause of TB in the study area. The findings are consistent with those of a study done in rural Uganda where witchcraft, hereditary factors, heavy labour, sharing of utensils and smoking were documented as the causes of TB [26]. Similarly, in a study done Tanzania, participants attributed TB to smoking, drinking alcohol, witchcraft and genetic factors [27]. The participants did not differentiate the cause of TB and the risk factors for disease development. While smoking and drinking alcohol may serve as risk factors for developing TB, they do not cause TB. Poverty and lack of awareness are considered the most important factors that increase the risk of exposure to TB while factors such as HIV/AIDS, smoking, drinking alcohol, malnutrition, increased susceptibility of infants and the elderly and increased virulence and/or increased dose of bacilli have been recognized as important contributors to the development of the disease and its epidemiological burden [7, 13, 28, 29]. The misperceptions about the cause of TB should be targeted through patient education and awareness creation in the community.

Similarly, the participants attributed casual contact such as greetings, eating together and sharing utensils with an infected person as a mode of TB transmission. As means of preventing TB, nearly all the participants reported the need for TB patients to have their own utensils which shouldn't be shared with the rest of the family. The findings of the current study are consistent with those of a recent study done among pastoralist communities in Ethiopia that showed significant knowledge gaps about the cause, signs and symptoms, mode of transmission, prevention, and treatment of TB among the community members [19]. While patients may have a general idea about TB, lack of knowledge about the cause, risk factors, mode of transmission and prevention may negatively affect the efforts geared to reducing the burden of TB in the community. The misconceptions about the cause and transmission affect the kind of preventive methods adopted by the community members. Community's understanding of the human transmission of infection by the TB patients is absolutely critical to the control of the disease. TB is a contagious, communicable disease that spreads to non-infected individuals when an infected patient expels droplets with TB microorganism to the surrounding environment as aerosol when coughing [30]. It is important for the TB patients to know the mode of transmission of TB as this can influence their behaviour such as cough etiquette, respiratory hygiene as well as seek early treatment, which is critical in preventing TB transmission [7, 16, 31]. In the current study, TB patients perceived TB as a communicable disease but had misconception on how the disease is transmitted. The findings resonate with those of Tolossa et al (2014) who found that while 80% of the participants knew TB was transmissible, 35.6% of the participants thought that sharing utensils with a TB patient was a route of transmission for the disease. In another study among a pastoral community in Ethiopia, participants felt that avoiding sharing utensils and sexual contact with TB patients would prevent the disease transmission [7]. These misconceptions are likely to misinform the community on the control and preventive measures they ought to institute.

## Conclusion

The study showed incorrect knowledge about TB among TB patients. Although the participants correctly perceived TB as a contagious disease they did not understand the correct cause and mode of transmission. There is a need to improve patient knowledge and awareness of TB. The current study is limited in that we only focused on patients and not the healthcare workers and the kind of patient education given at the TB clinics. Further studies to look into the kind of patient education given and its effectiveness in improving patients TB knowledge are recommended.

### What is known about this topic

- Previous studies have focused on community knowledge of tuberculosis and shown poor knowledge among study participants;
- Community members often have incorrect knowledge about the cause transmission and treatment of tuberculosis.

### What this study adds

- The current study focuses on TB knowledge and perceptions among Tuberculosis patients who are already on treatment. The patients have been in contact with health workers and should have received TB education as required by the TB program;
- The study shows the patients still exhibit TB knowledge gaps and recommend a need to heighten TB education by the TB program.
